# Supplementary material for: Predicting visual function by interpreting a neuronal wiring diagram
Source: Nature. 2024 Oct 2;634(8032):113–23. doi: 10.1038/s41586-024-07953-5 (PMC11446822; doi:10.1038/s41586-024-07953-5)
Supplement: Supplementary file 6 — CRF and ERF predictions for individual TmY4 and TmY9 cells. Analogous to Supplementary Data 3, but for TmY target types. Shown are the top four monosynaptic pathways, the strongest pathway passing through each of the top ten intermediary types (ranking from Extended Data Fig. 7), and the trisynaptic pathway Tm1–TmY–Dm3–TmY (see the section entitled Prediction of spatial normalization). [file 41586_2024_7953_MOESM6_ESM.zip › DataS4/TmY9qΓèÑ/720575940626925093.pdf]

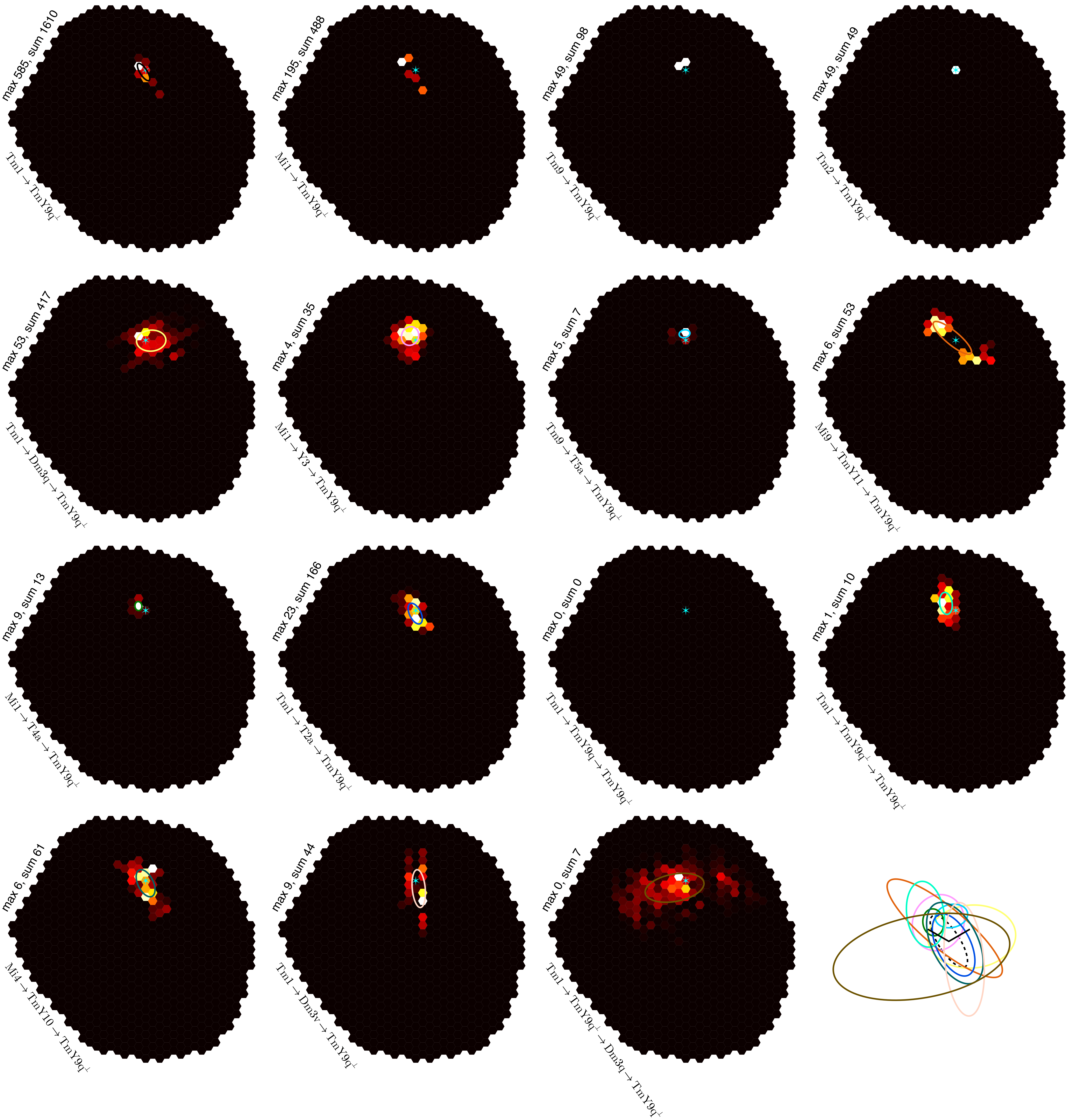
max 585, sum 1610
Tm1 → TmY9q⊥
max 195, sum 488
Mi1 → TmY9q⊥
max 49, sum 98
Tm9 → TmY9q⊥
max 49, sum 49
Tm2 → TmY9q⊥
max 53, sum 417
Tm1 → Dm3q → TmY9q⊥
max 4, sum 35
Mi1 → Y3 → TmY9q⊥
max 5, sum 7
Tm9 → T5a → TmY9q⊥
max 6, sum 53
Mi9 → TmY11 → TmY9q⊥
max 9, sum 13
Mi1 → T4a → TmY9q⊥
max 23, sum 166
Tm1 → T2a → TmY9q⊥
max 0, sum 0
Tm1 → TmY9q⊥ → TmY9q⊥
max 1, sum 10
Tm1 → TmY9q⊥ → TmY9q⊥
max 6, sum 61
Mi4 → TmY10 → TmY9q⊥
max 9, sum 44
Tm1 → Dm3v → TmY9q⊥
max 0, sum 7
Tm1 → TmY9q⊥ → Dm3q → TmY9q⊥
